# Tuberculin Skin Testing in Children

**DOI:** 10.3201/eid1205.050980

**Published:** 2006-05

**Authors:** Marina Reznik, Philip O. Ozuah

**Affiliations:** * Albert Einstein College of Medicine, Bronx, New York, USA

**Keywords:** Tuberculin skin testing, targeted, children, synopsis

## Abstract

In 1996, the American Academy of Pediatrics (AAP) recommended targeted tuberculin skin testing (TST) of children while discouraging routine TST of children without risk factors for tuberculosis (TB). Recent studies have provided evidence in support of the targeted TST and recommendations that favor risk assessment over universal screening with TST. While evidence for targeted TB testing exists and benefits of screening programs are clear, administrative logistics could be a greater issue. The challenge for public health and school officials is to develop a screening program that avoids stigmatization of the at-risk group. Until then, pediatric healthcare providers will continue to have a key role in identifying children at risk for latent TB infection by using the AAP-endorsed risk-assessment questionnaire and should screen children with TST only when >1 risk factor is present.

## Abstract 2

Skin testing is recommended for children at risk for this disease.

In the 1960s and 1970s, when tuberculosis (TB) infection rates in the United States were high, universal screening for TB was required for all children (*1*). Between the 1980s and early 1990s, in response to a new increase in incidence of TB cases in the United States (*2**–**4*), the American Academy of Pediatrics (AAP) recommended annual tuberculin testing for high-risk children such as blacks, Hispanics, the socioeconomically disadvantaged, and children living in neighborhoods where the disease rate was higher than the national average (*5*). In 1996, the AAP's committee on infectious diseases (*6*) issued updated guidelines that called for targeted tuberculin skin testing (TST) of children and discouraged universal testing of children who lack risk factors. More recently, these recommendations were reiterated by a joint statement of the American Thoracic Society, the Centers for Disease Control and Prevention (CDC), and the Infectious Diseases Society of America (*7*). We review the rationale and evidence in support of targeted TST in children and discuss some of the logistic aspects of instituting targeted screening programs.

## Who Is at Risk for TB Infection?

Targeted testing is intended to prevent progression of TB by identifying persons at risk for TB infection or disease who would benefit from treatment for latent TB infection (LTBI). Children at high risk for TB infection include contacts of persons with active TB; those who are foreign-born; those who travel to or have household visitors from a country with a high TB prevalence such as Mexico, the Philippines, Vietnam, India, and China (*8*); contacts with high-risk adults, including those who are homeless, incarcerated, infected with HIV, or intravenous drug users; and those with chronic conditions such as diabetes mellitus, renal failure, malnutrition, or other immunodeficiencies (*6**,**7*).

The rationale for targeted TB screening includes some of the following factors. The positive predictive value of any test, even one with high sensitivity and specificity, is extremely low in any population with low prevalence of the disease in question. Universal testing of such a population would lead to a low benefit-to-cost ratio. The sensitivity and specificity of TST are ≈90%, which results in a higher positive predictive value in high-prevalence populations (*9*). Among children with a 1% rate of TB infection, the positive predictive value is <10%. Thus, >90% of positive reactions are false positives (*10*). Since no test can distinguish false positives from true positives, all persons with positive TST results must be evaluated and treated. Falsely identifying TB in a child creates unnecessary cost for clinic visits, radiographs, treatment with isoniazid that has harmful side effects, family testing, and follow-up appointments. In addition, this false identification may cause anxiety as the physician and family try to determine the source of a nonexistent infection and create an ethical dilemma by labeling a child as infected with TB.

## Benefit of Targeted TST in Children

Previous studies have shown a benefit of the targeted TST in children (*11**–**13*). In a study of 2,169 children who had mandatory TST because they resided in a high-prevalence community, Ozuah et al. (*11*) found a low rate (0.5%) of TST reactivity. These findings support the revised AAP guidelines recommending targeted TST of children at high risk for TB. Cost-effectiveness of school-based targeted TST compared with universal screening of children in the United States, as well as in other countries, showed that targeted screening of schoolchildren was more cost effective than mass screening (*12**,**13*).

## Assessment of Risk Factors for LTBI in Children

Several recent studies have addressed the use of risk assessment to identify children who are likely to have reactive TST results (*14**–**18*). Although these studies assessed different populations, their findings were similar. Lobato et al. (*14*) conducted a case-control study in 953 children (<6 years of age) who had a TST read at public health clinics in California. Risk factors for a positive TST result (>10 mm) among the study population included >1 week foreign travel to a country with a high prevalence of TB within the past 12 months (odds ratio [OR] 3.9, 95% confidence interval [CI] 1.9–7.9) or a household visitor from such a country (OR 2.4, 95% CI 1.0–5.5).

Saiman et al. (*15*) conducted a multicenter, prospective, matched, case-control study in children (1–5 years of age) in New York who underwent TST by primary care providers during routine healthcare visits. Of 288 persons, 96 were cases (defined as persons with a TST result >10 mm and a normal chest radiograph) and 192 were age- and clinic-matched controls (defined as subjects with a TST result = 0 mm). This study identified several risk factors for LTBI in children: contact with an adult with TB (risk ratio [RR] 61.6, p = 0.0004), foreign birth (RR 9.2, p<0.0001), foreign travel (RR 7.5, p = 0.0002), or a family member with LTBI (RR 15.7, p<0.0001).

In a similar study, Besser et al. (*16*) identified risk factors for LTBI in children (<6 years of age) in San Diego, California, who received a TST as part of routine well-child care. Fifty-one persons with a TST result ­>10 mm and normal chest radiograph and 72 age-matched controls participated in the study. In this population, *Mycobacterium bovis* bacillus Calmette-Guérin (BCG) immunization (OR 53, 95% CI 13–224), a TST within 12 months (OR 24, 95% CI 1.7–347), or a relative with a positive TST result (OR 4.9, 95% CI 1.4–16.5) were risk factors for LTBI.

Froehlich et al. (*17*) conducted a prospective observational study to determine if a risk-assessment questionnaire could predict a positive TST result in a population of 31,926 children (1–18 years of age) in California. This study found that BCG immunization (OR 2.3, 95% CI 1.7–3.1), foreign birth (OR 8.6, 95% CI 6.2–12.1), living outside the United States (OR 2.1, 95% CI 1.5–2.9), Asian (OR 2.3, 95% CI 1.6–3.3) or Hispanic (OR 1.6, 95% CI 1.1–2.3) ethnicity, or contact with a household member with LTBI or TB (OR 1.5, 95% CI 1.1–2.0) were independent predictors of LTBI.

Ozuah et al. (*18*) conducted a prospective criterion standard study of 2,920 children (1–18 years of age) in the south Bronx, New York, to determine the sensitivity, specificity, and predictive validity of the New York City Department of Health (NYCDOH) risk-assessment questionnaire for identifying children who should receive a TST. Questionnaire risk factors for TB infection were contact with a case of TB, foreign birth or travel to a TB-endemic area, contact with adults at high risk for TB (those who are infected with HIV, homeless, incarcerated, and illicit drug users), and HIV infection in a child. Contact with an adult with TB (OR 91.7, 95% CI 32.3–260.7), foreign birth or foreign travel (OR 14.8, 95% CI 6.7–32.7), and contact with a high-risk adult (OR 6.5, 95% CI 2.4–17.5) were independent risk factors for a positive TST result. Results for the full NYCDOH questionnaire were sensitivity 85.2%, specificity 86%, negative predictive value 99.8%, positive predictive value 5.4%, and OR 35.2 (95% CI 12.1–102.4). The data were interpreted as demonstrating that the NYCDOH questionnaire was a valid instrument for identifying children for TST. Children with >1 identifiable risk factor were 35 times more likely to have a positive TST result.

## Screening Questionnaire for Risk Factors for LTBI

These studies have identified risk factors for LTBI in children. Based on these factors, a risk-assessment questionnaire was developed by the pediatric tuberculosis collaborative group to facilitate LTBI screening by pediatric healthcare providers (*19*). Pediatricians should ask the following questions when screening for risk factors of LTBI during the child's annual health maintenance visit (*19*). 1) Was your child born outside the United States? 2) Has your child traveled outside the United States? 3) Has your child been exposed to anyone with TB? 4) Does your child have close contact with a person who has had a positive TB skin test result? 5) Does your child spend time with anyone who has been in jail or a shelter, uses illegal drugs, or has HIV? 6) Has your child drunk raw milk or eaten unpasteurized cheese? 7) Does your child have a household member who was born outside the United States? 8) Does your child have a household member who has traveled outside the United States? A child or adolescent should be tested with TST only if >1 risk factor is present.

## Challenges with Targeted Screening and LTBI Treatment Adherence

Despite the revised AAP recommendations for targeted TST and evidence for use of risk assessment, putting these guidelines into practice have presented some challenges. In 1996, the New York City Health Code was amended to require TST of only new entrants to secondary schools to reduce unnecessary screening of primary schoolchildren at low risk for LTBI. A study by Gounder et al. (*20*) assessed adherence to this revised health policy change and showed that the proportion of new entrants into New York City's primary schools who were tested remained virtually unchanged after implementation of the health code amendment to discontinue testing of these children. In addition, older children who were more likely to be born in countries with high TB incidence and were at risk for LTBI were not tested.

Lack of clinician adherence to the LTBI screening guidelines has been shown in another study. Hsu et al. (*21*) found that most adolescents identified by risk-assessment questionnaire to be at risk for LTBI in 3 Boston schools were not adequately screened for TB infection. These studies show the necessity of programs to improve healthcare provider knowledge and acceptance of targeted TB screening guidelines. Research studies to assess the effect of such educational programs for clinicians on the targeted TB screening outcomes are needed. Future studies should also be conducted to compare the effectiveness of routine TST for all new high school entrants versus the use of the risk-assessment questionnaire in different populations.

Children diagnosed with LTBI must complete the prescribed regimen of isoniazid to maximize the protective effects of therapy. However, patient adherence to treatment for LTBI is low. Prevous studies have assessed different strategies to improve adherence to LTBI treatment (*22**–**25*). Morisky et al. (*22*) determined the effects of educational strategies to improve treatment of LTBI among adolescents in Los Angeles by randomly assigning them to a peer-counseling group, a group that received incentives, a combination of peer counseling and incentives, and a usual-care group. They found no difference in the rates of completion of LTBI treatment among the 4 groups.

Cass et al. (*23*) evaluated the effectiveness of a behavioral intervention, the Treasure Chest, to increase adherence to LTBI therapy in children. Each person received a monthly calendar with stickers and instructed to place a sticker on each day the medication was taken. When the completed calendar was returned, the child was allowed to select a toy from the Treasure Chest as a reward. Children who participated in the Treasure Chest program were 2.4 times more likely to complete therapy than those who did not. Several studies have also reported a significantly higher rate of completion of LTBI treatment among those high school students receiving directly observed therapy (*24**,**25*). Future studies should evaluate measures of adherence to LTBI therapy and other methods (educational efforts and various incentives) to improve adherence among different pediatric age groups and populations.

## Logistic Aspects of Targeted Screening Programs

While the evidence for targeted TB testing exists and benefits of screening programs are clear, administrative logistics are of greater concern. The challenge for public health and school officials will be to develop a screening program that would avoid stigmatization of the at-risk group. One way to reduce stigmatization of the targeted group of children is to leave identification and screening of these children in the hands of their primary care providers. In New York City, every school year a medical information form that includes TST results is required for each currently or newly enrolled student (*20*). This form has recently been revised to reflect the targeted TB screening guidelines; however, not all schools have the updated forms.

To avoid stigmatization, targeted screening could also be accomplished by involving community organizations and local clinics that serve recent immigrants or the homeless population. For example, using community health workers who are members of the targeted communities may help eliminate language and cultural barriers in populations who are difficult to reach and screen for TB. Future studies are needed to assess the effectiveness of community health workers in improving the targeted screening of at-risk populations.

## Conclusions

Several recent studies have shown the benefit of targeted TST and validated the use of risk-assessment questionnaires to identify children at increased risk for acquiring TB (*11**–**18*). These studies provide evidence in support of the targeted TST and recommendations favoring risk assessment over universal screening with a TST. Targeted TST and proper management of children with LTBI are essential components of the TB-elimination strategy promoted by the United States Public Health Service Advisory Council on the Elimination of Tuberculosis (*26*). Although targeted screening for LTBI in pediatric populations remains the current recommendation of CDC and AAP, clinician nonadherence to these guidelines results in overtesting children at low risk for LTBI and undertesting children at high risk for LTBI. In addition, the logistic issues with targeted screening programs are important. Public health measures must identify but not discriminate against high-risk populations. However, in practice, pediatric healthcare providers will continue to have a key role in identifying children at risk for LTBI. Thus, they should be familiar with risk factors for LTBI and screen children with TST only when >1 risk factor is present.
